# Supplementary material for: Microhomology Selection for Microhomology Mediated End Joining in Saccharomyces cerevisiae
Source: Genes (Basel). 2019 Apr 8;10(4):284. doi: 10.3390/genes10040284 (PMC6523938; doi:10.3390/genes10040284)
Supplement: Supplementary file 1 [file genes-10-00284-s001.pdf]

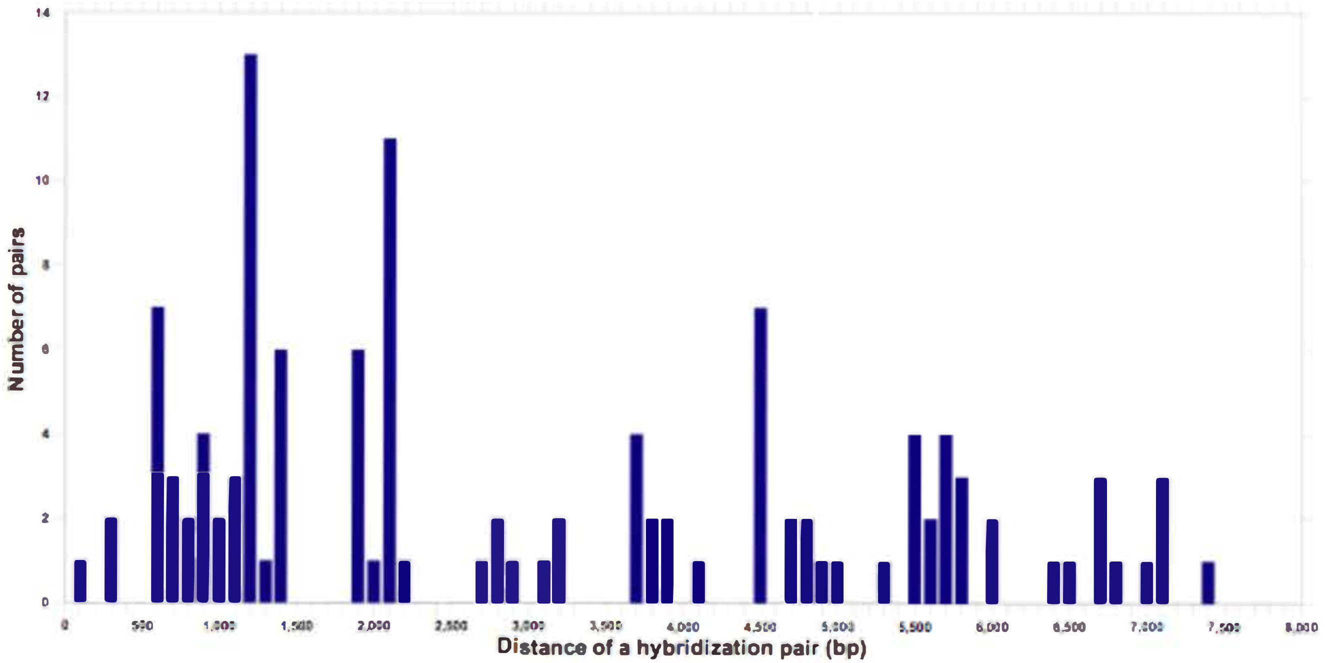

Figure S1. The distributionof 12-mer hybridization pairs in terms of their spacing; distanceof a pair is calculate in basepairs from HO cut site. 119 hybridization pairs out of more than 2 million pairs are selected by applying criteria of match score > 0.5 and normalized energy score > 0.5.
